# PP49 Prescribing Trends And Economic Impact Of Somatostatin Analogues In Acromegaly: A Threat To Brazil’s Public Health System Sustainability

**DOI:** 10.1017/S0266462324002204

**Published:** 2025-01-07

**Authors:** Lara Benigno Porto Dantas, Fellipe Miguel Mendes de Farias, Thizzah Cecília de Sousa Alves da Silva, Luciana Ansaneli Naves, Ivan Ricardo Zimmermann

## Abstract

**Introduction:**

First-generation somatostatin analogues (SSA) are indicated as a first-line medical treatment for acromegaly in patients with persistent disease after surgery or who are not eligible for surgery. These drugs are the largest contributor to the direct medical cost of acromegaly management worldwide. We analyze the patterns of prescription and costs of SSA for the treatment of acromegaly in Brazil.

**Methods:**

The first-generation SSA, octreotide LAR (OCT-LAR) and lanreotide autogel (LAN-ATG), are available in Brazil to treat acromegaly through the Specialized Component of Pharmaceutical Assistance policy. The public records of the nationwide database (DATASUS) were accessed to identify the drug use patterns during the year 2022. The current values of the drug acquisition costs of each medication in 2022 were consulted at the national prices database (BPS). Results were converted in purchase power parity (PPP) dollars according to the World Bank rates.

**Results:**

The acquisition cost of each octreotide (OCT-LAR) ampoule was USD492.33 (10 mg), USD537.04 (20 mg), and USD703.79 (30 mg); for lanreotide (LAN-ATG), each ampoule was USD394.74 for the 60 mg dosage, and USD418.48 for the 90 mg and 120 mg dosages. The average annual cost of a monthly 30 mg dosage of OCT-LAR would be estimated as USD8,445,48 against an average annual cost of USD5,021.76 for a monthly 120 mg dosage of LAN-ATG. Thus, we estimate that USD15,520,747.59 was spent on first-generation SSA to treat acromegaly in Brazil’s Unified Health System (SUS) in the year 2022; of this, 79 percent was attributed to OCT-LAR.

**Conclusions:**

Life-long treatment with SSA is related with a high economic burden in Brazil. There is a predominance of OCT-LAR prescription, where expanding the use of LAN-ATG may help reduce costs to the SUS. Nevertheless, studies and investments in other treatments, such as pituitary surgery and radiotherapy access, at a national level are also essential to improve acromegaly treatment costs.

